# Genetic analysis of maximum cigarette-use phenotypes

**DOI:** 10.1186/1471-2156-4-S1-S105

**Published:** 2003-12-31

**Authors:** Nancy L Saccone, Rosalind J Neuman, Scott F Saccone, John P Rice

**Affiliations:** 1 Department of Genetics, Washington University School of Medicine, St. Louis, Missouri, USA; 2 Department of Psychiatry, Washington University School of Medicine, St. Louis, Missouri, USA

## Abstract

**Background:**

Using the Framingham Heart Study data set provided for Genetic Analysis Workshop 13, we defined the cigarette-use phenotype *M *for smokers to be the maximum number of cigarettes-per-day (MAXCIG) reported over the longitudinal course of the study. Adjustments were made for the significant covariates of gender and year of birth, and sib-pair based linkage analysis was performed.

**Results:**

The primary analyses, in which individuals with MAXCIG = 0 were considered to have missing phenotype, resulted in modest linkage evidence, with LOD scores over 1 on chromosomes 5, 9, 13, 14, and 22.

**Conclusions:**

While the results reported here do not indicate definitive evidence for linkage to specific chromosomal regions, future studies may find it useful to include direct assessments of maximum and quantitative cigarette use. In defining and analyzing quantitative or "maximum use" phenotypes, the choice of how to handle individuals with MAXCIG = 0, or alternatively, individuals who are substance-naive, is a crucial one for genetic studies of nicotine and other substance use. In this study, the linkage results vary greatly depending on whether or not these "unexposed" individuals are included in the analyses.

## Background

Cigarette smoking is a leading cause of premature death and is a serious public health concern. Past studies of smoking behavior have considered a variety of smoking phenotypes and found that while smoking-related traits are complex, there is evidence for significant genetic influences on smoking behavior [1,2].

The Framingham Heart Study data set provided for Genetic Analysis Workshop 13 (GAW13) includes longitudinal data on daily cigarette use. In the past the Framingham data have been used to study important cigarette smoking patterns such as cessation and resumption [3]. Our focus here was to define cigarette-use phenotypes that have potential to be useful in genetic analyses. Reports from the Collaborative Study on the Genetics of Alcoholism (COGA) have shown that the "maximum number of drinks ever consumed in a 24 hour period" is a useful phenotype for discovering potential genetic influences on alcohol dependence [4].

## Methods

The cigarette smoking data consist of the number of cigarettes smoked per day during periods of use in the year prior to each exam. We have defined cigarette-use phenotypes based on these available data. However, as the exams were (generally) 2 years apart for the original cohort and 4 years apart for the offspring cohort, it is possible that periods of smoking behavior could have been missed. Furthermore, no information was available on lifetime smoking, the duration of regular smoking over the past year, or whether the subject was still currently smoking at each exam.

We defined a maximum number of cigarettes phenotype *M*, or MAXCIG, as the maximum cigarettes-per-day reported over all the available exams (up to 18 exams with cigarette data for the original cohort and 5 exams for the offspring cohort). For the purposes of the descriptive results below, the term "smokers" refers to individuals with *M *> 0, and "non-smokers" refers to individuals with *M *= 0. However, it is important to note that *M *is not the same as true lifetime maximum use, which cannot be determined from the longitudinal data provided.

For genetic analyses, the primary phenotype was taken to be the maximum number of cigarettes phenotype where individuals with *M *= 0 were excluded by recording their phenotype as "unknown." From past experience with alcohol phenotypes we expect that defining substance-naive individuals to have unknown phenotype is most appropriate, as individuals who have not been exposed to a substance have unknown response; however, we also performed parallel analyses that included *M *= 0 individuals for comparison. When *M *= 0 individuals are included, we obtained 714 nuclear families with at least two phenotyped offspring, providing 1545 non-independent phenotyped and genotyped sib pairs. For the primary phenotype (*M *= 0 considered unknown), there are 412 such nuclear families, containing 621 non-independent phenotyped and genotyped sib pairs.

Linear regression (using SAS, SAS Institute, Cary, NC) was used to correct the primary maximum-cigarette phenotype for the significant covariate of gender in the initial linkage analyses. Additional regression models adjusted for both gender and year of birth (with linear and quadratic terms and appropriate rescaling), both with and without interaction terms. Each of the resulting phenotypes was used for linkage analysis. Note that a similar regression adjustment is not as appropriate in the case where *M *= 0 individuals are included.

The original and offspring cohorts have different time intervals between exams; this difference could lead to systematic differences in the resulting smoking phenotypes. Thus we have compared maximum cigarette use when sampling at 2-versus 4-year intervals in the original cohort to examine whether this difference in time interval significantly affects the resulting phenotype.

Multi-point linkage analysis was carried out on all sib pairs (*n*(*n*-1)/2 pairs for a sibship of size *n*) using Haseman-Elston regression as implemented in MAPMAKER/SIBS [5].

We also examined descriptive birth cohort effects on *M*. Since the exact year of birth was not directly given in the data, we approximated year of birth as follows: for the original cohort the age at first exam (age 1) was subtracted from the starting year of the study (1948); for the offspring cohort, age at first exam was subtracted from 1971, the starting year for the offspring recruitment. Using these definitions, there were only five men and eight women in the sample who were born in the 1960s (and none later), so binned cohorts were defined by decades with the youngest cohort having a birth year in the 1950s or later.

## Results

In the full sample of original cohort and offspring cohort, *M *ranged from 0 to 95. For this full sample, among smokers the mean for M was 20.4 for females and 27.6 for males.

We compared descriptive results for *M *for the original cohort to the alternative phenotype *M*_*o *_obtained from 4-year exam intervals rather than 2-year intervals. Specifically, *M*_*o *_is the maximum cigarettes-per-day reported over all available odd-numbered exams (corresponding to exam intervals of 4 years with 6 years between the first two exams). Among men, 30.8% reported *M *= 0 versus 34.6% who reported *M*_*o *_= 0. Among women, 52.8% reported *M *= 0 versus 55.0% with *M*_*o *_= 0. Among smokers, a t-test showed no significant difference between the means for *M *and *M*_*o *_in either gender. Hence the difference in the frequency of exams between the two cohorts does not appear to be a serious concern in defining *M*.

Sib-pair correlations were significant for *M*, consistent with a familial trait. The correlations were 0.25 in male pairs (*p *< 0.0001), 0.33 (*p *< 0.0001) in female pairs, and 0.13 (*p *= 0.002) in male-female pairs. Lower correlation in male-female pairs compared to same-sex pairs has similarly been observed for maximum alcohol consumption traits [4].

Maximum cigarette use levels by gender and birth cohort are presented in Table 1. In the regression analyses to adjust *M *(with *M *= 0 recorded as missing) for covariate effects, gender was highly significant (*F *= 115, *p *< 0.0001). Year of birth, tested with two degrees of freedom for the linear and quadratic terms, was also significant when added to the model with gender (*F *= 25, *p *< 0.0001). Finally, including interaction terms was also significant at the 0.05 level (*F *= 3.61, *p *= 0.027); this is consistent with the pattern of effects in Table 1, which indicates that the effect of birth cohort is somewhat different for males than for females.

Linkage results are summarized in Table 2. Multi-point linkage analyses of *M *corrected for gender (and *M *= 0 recorded as missing) resulted in LOD scores of 1.10 at 200 cM on chromosome 5, 1.67 at 173 cM on chromosome 9, 1.18 at 41 cM on chromosome 13, 1.29 at 87 cM on chromosome 14, and 1.04 at 3 cM on chromosome 22. Results for birth-year-adjusted analyses were very similar, with a slight increase in LOD score for chromosome 9 (1.77 for the full-model adjusted trait).

When nonsmokers were included in the analysis, linkage results were substantially different from those of the primary analyses above: none of the above regions gave a LOD above 1. Instead, LODs > 1 occurred on chromosomes 6, 8, 15, 17, and 20 (Table 2).

## Discussion and Conclusions

The linkage results for these maximum use phenotypes are modest. These phenotypes were constructed from the available longitudinal data. The motivation for defining *M *was that it provided a possible cigarette-use analog to "maximum number of drinks in 24 hours"; linkage analyses of this maximum drinks phenotype in data from the Collaborative Study on the Genetics of Alcoholism (COGA) indicated evidence for linkage to the region of the alcohol dehydrogenase gene cluster on chromosome 4 [4].

Additional corrections for age effects in addition to sex effects did not substantially affect the linkage analysis results, even though age is clearly a significant covariate for the maximum cigarette use phenotype studied. This minimal impact on results could be due to the fact that sib-pair based linkage analysis was performed; as siblings tend to be similar in age, age-adjustment would tend to have lesser effect on trait differences within pairs, although scaling differences between sib pairs of different cohorts still could have a meaningful effect.

There were substantial differences in results of the linkage analyses for which *M *= 0 individuals were set to missing compared with those for which *M *= 0 individuals were included. The two phenotype definitions provide very different sample sizes, and while there are a similar number of LOD scores over 1 for each phenotype, none occur in the same regions (across the phenotypes). There are at least two reasons why these differences in results may be expected. First, *M *= 0 is being used as a proxy to recognize substance-naïve individuals, some of whom might nevertheless be susceptible to heavy cigarette use if suitably exposed; thus inclusion of these individuals with a trait value of zero could substantially alter the phenotype, in part from an effect similar to that of misdiagnosis, with individuals sharing genetic susceptibility having erroneously dissimilar phenotype. Second, we see from Table 1 that the percentage of individuals with *M *= 0 is quite high, and including these subjects with *M *= 0 not only increases the sample size dramatically, but also forces a trait distribution with properties very different from those of the distribution for *M *> 0 individuals only. Specifically, there is a floor effect that is problematic for the distributional requirements of regression analysis.

While Haseman-Elston regression is often described as a "distribution-free" linkage method, this property is usually invoked when pointing out protection from false positives; it is worth noting that changes in and rescaling of a trait's distribution will affect the power of an analysis to detect a particular effect. We used classical Haseman-Elston regression for our primary analyses due to concerns about the distributions of the quantitative traits studied. Besides Haseman-Elston regression, MAPMAKER/SIBS can also be used to perform ML (maximum likelihood) quantitative trait locus variance estimation on sib-pair data. However, this method makes a necessary assumption of normality in the distribution for sib pairs within each identity-by-descent (IBD) class at a locus. ML variance estimation of the sample with *M *= 0 set to missing did result in LOD scores that were at least partially supportive of the Haseman-Elston findings (e.g., in sex-adjusted analyses, LOD scores over 1 on chromosome 5 and chromosome 9 occurred in the same regions in Table 2, in addition to LODs over 1 on chromosomes 3 and 11). However, when *M *= 0 subjects were included, results peaked repeatedly throughout the genome, even when no peaks were observed with Haseman-Elston regression. It seems advisable to consider these latter ML variance results (which included LOD scores above 5 on chromosomes 1, 11 and 20 in sex-adjusted analyses) not meaningful due to the apparent violation of distributional assumptions.

It should be noted that the maximum cigarette phenotype *M *studied here would be expected to differ from phenotypes obtained from direct questions such as "How many cigarettes per day did you smoke during your heaviest period of use" or "What is the largest number of cigarettes you have ever smoked in a 24-hour period". Maximum or quantitative cigarette-use phenotypes obtained from direct questions such as these may prove useful in other studies. It also appears useful to include questions that would permit direct identification of substance-naive or minimally exposed individuals.

## Figures and Tables

**Table 1:** Maximum cigarette use (%) by birth cohort^A^

**Born:**	**Before 1920**	**1910s**	**1920s**	**1930s**	**1940s**	**1950s or later**
Men						
*M *= 0	38.67	21.11	27.21	28.19	38.70	52.67
1 *≤ M *< 5	2.72	2.22	4.76	1.32	2.74	5.34
5 *≤ M *< 10	3.32	1.48	1.36	0.88	2.40	2.29
10 *≤ M *< 20	8.46	4.81	9.52	9.25	11.30	8.40
20 *≤ M *< 30	27.19	24.07	21.77	24.23	17.81	11.45
30 *≤ M *< 40	7.25	17.78	10.88	16.74	12.67	9.92
40 *≤ M *< 50	9.06	20.00	12.93	13.66	9.59	7.63
50 *≤ M *< 60	2.42	4.81	5.44	2.20	2.40	1.53
60 *≤ M *< 70	0.91	3.70	4.76	3.08	2.40	0.76
70 *≤ M *< 80	0	0	0	0	0	0
*M *≥ 80	0	0	1.36	0.44	0	0
Total *N*	337	270	147	227	292	131
Women						
*M *= 0	62.91	41.23	42.01	38.16	39.20	41.59
1 *≤ M *< 5	4.45	4.31	5.33	2.63	4.65	12.39
5 *≤ M *< 10	4.75	8.00	5.92	3.51	6.98	5.31
10 *≤ M *< 20	5.93	12.92	7.10	13.60	11.30	9.73
20 *≤ M *< 30	13.06	18.77	23.08	19.30	17.94	22.12
30 *≤ M *< 40	5.93	6.46	8.28	12.72	11.30	5.31
40 *≤ M *< 50	2.37	6.77	7.10	8.33	6.64	3.54
50 *≤ M *< 60	0.59	0.92	0.59	0.88	1.33	0
60 *≤ M *< 70	0	0.62	0.59	0.88	0.66	0
70 *≤ M *< 80	0	0	0	0	0	0
*M *≥ 80	0	0	0	0	0	0
Total *N*	337	325	169	228	301	113

^A^M = maximum cigarette use over all available examinations.

**Table 2:** Linkage results from multi-point Haseman-Elston regression.^A^

		Covariates					
		
		Sex		Sex, YOB^B^, YOB^2^		Sex, YOB, YOB^2^, interactions	
				
Chr	Marker(s)	cM	LOD	cM	LOD	cM	LOD
*M *= 0 coded as missing							
5	Mfd154-164xb8	200	1.10	200	1.08	200	1.02
9	308vb1-ATA59H06	173	1.67	172	1.78	172	1.77
13	GATA86H01-GATA6B07	41	1.18	41	1.11	41	1.28
13	GATA64F08-GATA7G10	-	-	-	-	66	1.16
14	GGAA4A12-*M*fd190	87	1.29	88	1.37	88	1.29
22	217xf4	3	1.04	3	1.05	3	1.06
*M *= 0 included							
6	242zg5	182	1.47	179	1.27	181	1.49
8	ATA19G07-GAAT1A4	115	2.31	116	2.02	115	2.05
15	GATA85D02	-	-	78	1.22	78	1.10
17	ATA78D02-GATA185H04	43	1.02	-	-	-	-
20	GATA47F05-321xd1	81	1.15	81	1.21	80	1.13

^A^Regions with peak LOD > 1 for at least one phenotype definition are shown. ^B^YOB, year of birth (as described in text).
